# Stationary External Electric Field—Mimicking the Solvent Effect on the Ground-State Tautomerism and Excited-State Proton Transfer in 8-(Benzo[d]thiazol-2-yl)quinolin-7-ol

**DOI:** 10.3390/molecules29153506

**Published:** 2024-07-26

**Authors:** Lidia Zaharieva, Ivan Angelov, Liudmil Antonov

**Affiliations:** Institute of Electronics, Bulgarian Academy of Sciences, 1784 Sofia, Bulgaria; zaharievalidia@gmail.com (L.Z.); ipangelov@gmail.com (I.A.)

**Keywords:** tautomerism, switching, DFT, external electric field, proton transfer, solvent effect

## Abstract

The effect of the external electric field on the ground-state tautomerism in 8-(benzo[d]thiazol-2-yl)quinolin-7-ol has been studied by using density functional theory. The compound exists as an enol tautomer (off state) and under the influence of the external electric field a long-range intramolecular proton transfer can occur, placing the tautomeric proton at the quinolyl nitrogen atom (on state). This is a result of the much higher dipole moment of the end keto tautomer and indicates that the external electric field can be used to mimic the implicit solvent effect in tautomeric systems. In the excited state, the further stabilization of the most polar on state leads to a situation when the excited-state intramolecular proton transfer becomes impossible, limiting the intramolecular rotation to the conical intersection region.

## 1. Introduction

The importance of the solvent on relative stabilization of the tautomers has been known since the first days of tautomeric research [1]. In 1896, Claisen in his review [2] defined the major factors that determine the relative stability of the tautomers—the structure (vitally important), the temperature and, if dissolved, the solvent. The most typical and easiest experimental test to prove a moveable tautomeric equilibrium is to measure the absorption spectra of the compound in various solvents [3], and if they differ (and other processes are excluded) the compound exhibits tautomeric forms with near stability, which can be stabilized/destabilized by changing the solvent. The effect of the solvent can be considered at two levels [3,4,5,6,7,8,9]: the implicit solvation, when the solvent acts as a continuum stabilizing/destabilizing the individual tautomers depending on their polarity, and the explicit solvation, where the stabilizing effect of the continuum on a given tautomer can be enlarged, reduced or even nulled depending of the ability to form stable solute–solvent complexes (mainly based on forming intermolecular hydrogen bonding) and their relative strength. In other words, the explicit solvation depends on the ability of the solute and the solvent molecules to interact in a “specific” way, while the implicit effect always exists.

The use of external electric field (EEF) can be considered as an alternative that mimics the implicit solvation [10], based on the fact that the implicit solvent effect is mainly based on stabilization due to different dipole moments. In this way, the EEF, being applied, can give another option to control the tautomeric state in a single solvent without changing the solvent by itself. There are a few examples where the use of EEF with different strengths can potentially change the tautomeric state of the system. Unfortunately, currently there is no reliable experimental evidence that this hypothesis works, and all studies up to now present theoretical results.

**Scheme 1:**
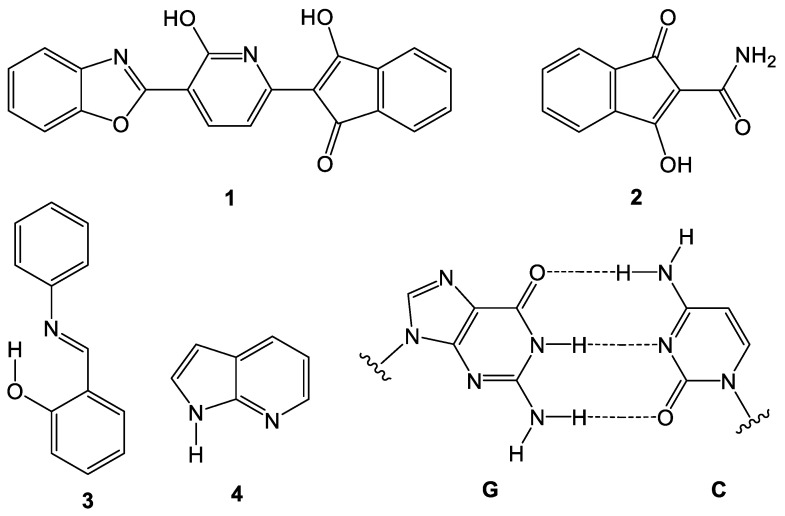
Compounds for which tautomerism has been studied under EEF up to now.

The tautomeric equilibrium of **1** and **2** (Scheme 1) under the influence of EEFs was studied by Enchev et al. [11,12]. The first one is a model system consisting of benzoxazolyl, hydroxypyridyl and indanedionyl fragments, being connected by single bonds, for which MP2 correlated ab initio calculations were used. The results show that the field’s strength and polarity can stabilize different tautomers, based on intramolecular proton transfer, alter the HOMO-LUMO gaps and shift absorption wavelengths. The second study investigates 2-carbamido-1,3-indandione [12], where SCS-MP2 calculations suggest that an electric field as low as 0.0003 a.u. (0.001543 V/Å) can invert the tautomeric equilibrium and stabilize 3-hydroxy-1-oxo-1H-indene-2-carboxamide **2**. It should be taken into account that these two tautomers are approximately the same stability, according to the used theoretical model, which makes the shift of the equilibrium relatively easy. Using ab initio quantum chemical calculations and on-the-fly dynamics simulations, Jankowska et al. proposed a novel EEF-driven molecular switch based on intramolecular proton transfer in salicylidene aniline **3** [13]. A stabilization of the keto form is observed when a field (0.01 a.u., corresponding to 0.0514 V/Å) is applied along the main axis of the molecule. Higher field strengths remove the energy barrier of the enol-to-keto transition, making the keto tautomer even more stable. Li et al. used (TD)-DFT calculations to show the effect of the static electric fields on water-assisted proton transfer in 7-azaindole **4** [14]. The EFs, applied in various directions, significantly impact the reaction energetics or the synchronism/asynchronism of intermolecular double-proton transfer. Fields aligned with the net proton transfer direction stabilize specific tautomers, tuning the thermodynamic balance and barrier heights in both ground and excited states. By using DFT calculations, Arabi et al. investigated the Watson–Crick guanin–cytosine (**GC**, see Scheme 1) base pair under the influence of EFs [15,16]. Strong fields ~2.57 × 10^7^ V/cm (≈0.027 a.u.) applied along the +x direction (**G** to **C**) accelerate the reaction and favor the formation of rare tautomers. However, weaker fields or those in the -x direction maintain DNA fidelity.

**Scheme 2:**
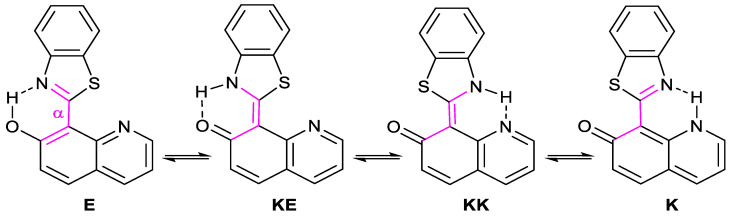
Ground-state tautomeric forms of **HQBT**. The angle of rotation of the rotor (benzothiazole) against the stator (7-hygroxy quinoline) is indicated in pink as a twisting angle α.

**Figure 1:**
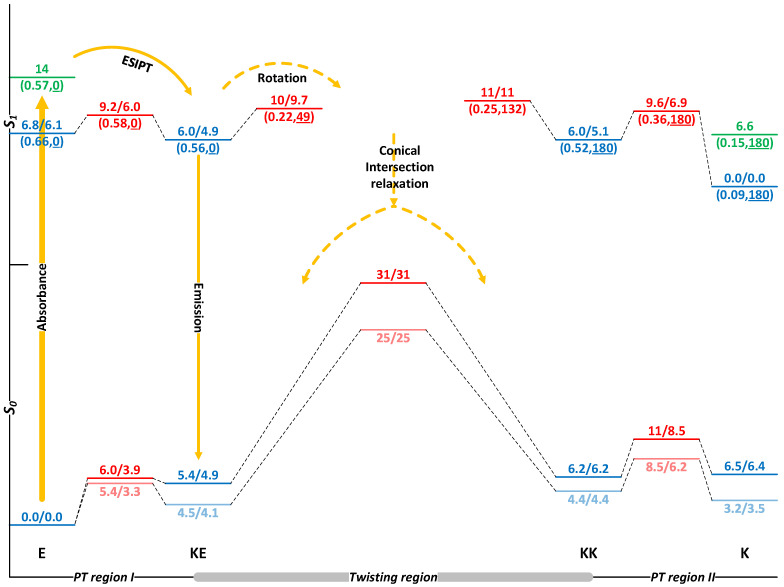
Potential energy surface (PES) of **HQBT** in toluene in ground and first singlet excited state. The stationary forms are indicated in blue, the transition states—in red and the Frank–Condon state—in green. The numerical values present relative energy DE/relative Gibbs free energy DG and are given in kcal/mol units. The ground-state PES in acetonitrile is presented with the same colors but in transparency. In the excited state, the oscillator strength and the angle of rotation of the rotor against the stator are given in brackets, the latter is underlined.

In fact, the tautomeric compounds can be considered as proton exchanging switching systems in which the switching process can be triggered by changing the solvent. This is easy to do in laboratory conditions, but it is technologically inconvenient if a real switching application is sought. Very recently, we have described a highly efficient tautomeric switching system [17], namely 8-(benzo[d]thiazol-2-yl)quinolin-7-ol (**HQBT**, Scheme 2), where the proton is transferred from the oxygen of the 7-hydroxy quinoline to the nitrogen upon irradiation in a series of consecutive steps (Scheme 2). The efficiency of the process strongly depends on the relative stability of the ground-state end tautomeric forms **E** (off state) and **K** (on state) as well as on the intermediate **KE** and **KK**. Actually, the switching of this compound very clearly shows the importance of the solvent. In toluene, the **KK** and **K** tautomers are similar in energy, which does not allow for the achievement of clean switching to **K** (Figure 1), while the strong solvent stabilization of the more polar **K**-form leads to a clean switching with >95% efficiency in acetonitrile. 

This opens an interesting question—is it possible to find additional stimulus that can make a clean switching in toluene? This is a general question not only for this particular compound but for any equilibrium system in general. Therefore, in the current study we will theoretically investigate the influence of the EEF on the ground and excited-state tautomerism of **HQBT** in toluene and how the photoswithching mechanism, based on long-range intramolecular proton transfer (LRIPT), is affected by the changes. The compound has been selected because its switching mechanism has been experimentally studied in detail and there is a theoretical methodology which correctly describes the experimental data [17]. The same (TD)-DFT methodology is used in this paper. To the best of our knowledge, this is the first study on the EEF effect as a mimic of the solvent polarity change in switching systems with LRIPT.

## 2. Results and Discussion

### 2.1. Switching of HQBT in the Absence of EEF

The processes of tautomerism and photoswitching of **HQBT** are sketched in Figure 1. According to the experimental data, the compound exists as a single **E** tautomer in solution giving absorbance at around 360 nm [17]. The theoretical data also predict a substantial stability of the enol form against the other possible three tautomers. Emission is detected at around 440 nm, corresponding to a fast ESIPT to KE* and consecutive relaxation from **KE** to **E** by ground-state intramolecular PT (GSIPT). If the Gibbs free energies are taken into account, both E* to KE* ESIPT and **KE** to **E** GSIPT can be considered as barrierless, see Figure 1. The same is suggested for the ESIPT by using coupled cluster calculations [17]. In other words, in the absence of irradiation, the processes are located in the *PT region I* as indicated in Figure 1. 

**Figure 2:**
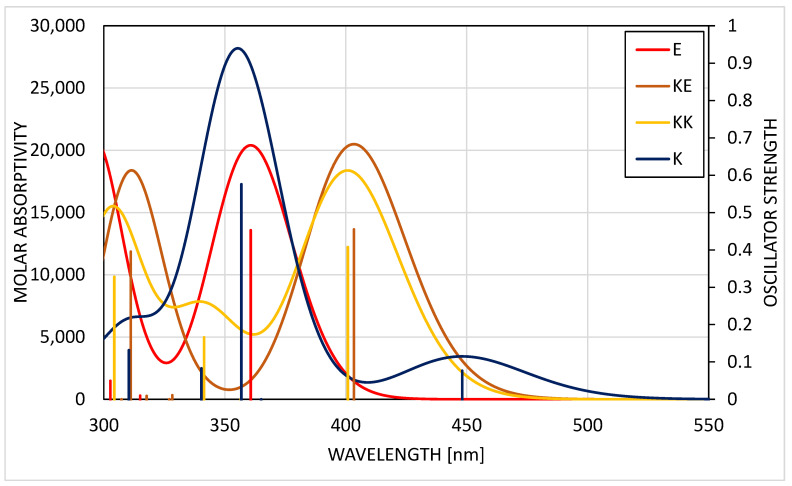
Theoretically predicted spectra of the ground-state tautomers of **HQBT** in toluene given as simulated absorption curves (**left**) and single transitions with corresponding oscillator strengths (**right**).

The intramolecular hydrogen bonding is broken upon irradiation in a substantial part of the existing KE* tautomers, and as a result of a rotation around the double bond, connecting benzothiazole and quinoline rings, the excited particles relax to the ground state through a conical intersection (*twisting region*, Figure 1). The twist of the benzothiazole rotor roughly corresponds to the rings being perpendicular, which leads to simultaneous population of the ground-state **KE** and **KK**. In the frame of the *PT region I*, the initial enol is restored, while the *PT region II* leads to obtainment of **K**. The slow thermal relaxation of **K** back to **E**, compared to the very fast ESIPT/GSIPT cycle E* → KE* → **KE** → **E** in the *PT region I*, allows for the accumulation of **K** (the on state), while the irradiation is turned on. The appearance and accumulation of **K** can be monitored as red shifted absorption at 460 nm, as suggested by the simulated spectra shown in Figure 2. A new emission coming from the **K**-form is observed around 560 nm, most probably originating from K* [17]. As seen from Figure 1 the excited **K** is low in energy, which makes the ESIPT to KK* less possible. The excited-state DLPNO-STEOM-CCSD calculations also suggest the relative stabilization of K* in respect to KK* [17]. The accumulated **K** relaxes thermally to **E** when the irradiation is switched off. 

### 2.2. The Effect of EEF in Solution

**Figure 3:**
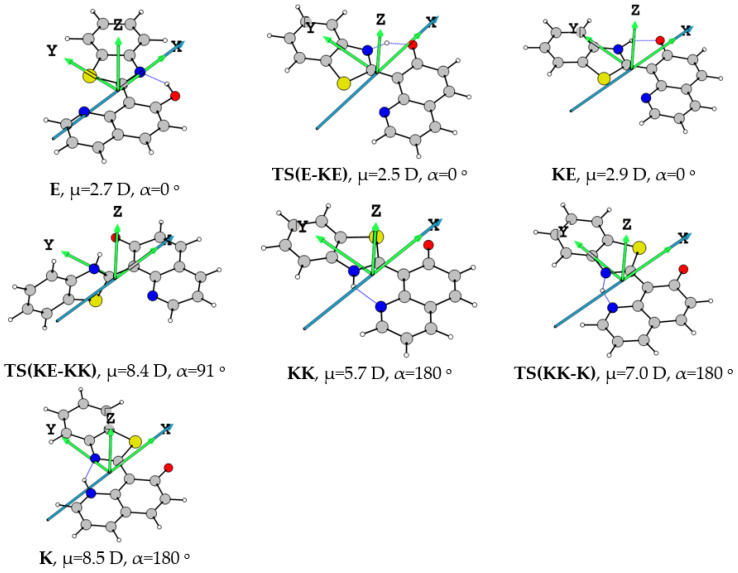
Direction of the dipole moment vectors (in light blue) of the ground-state tautomeric forms and transition states of **HQBT** in respect to the local coordinate system (in green). The atoms are given as follows: O (red), N (blue), S (yellow), C (grey), H (white). The values of the dipole moments and twisting angle (see Scheme 2) are given for information.

In order to facilitate the effect of the homogeneous EEF on the ground and excited-state PES of **HQBT**, some important details should be taken into account. As noticed before [10,18], a substantial part of the applied field strength is initially used to orient the solvent molecules. Therefore, to minimize this effect, toluene is used as a solvent in the current study. The toluene is low polar (μ = 0.31 D), neither a proton acceptor nor a proton donor solvent, from one side, which allows for the neglect of the solvent component of the EEF effect [19] in respect to the much more polar **HQBT** tautomers (see Figure 3). On the other side, previous experimental data for the ground-state tautomerism and switching of this compound [17], confirming the agreement between the theoretical DFT approach, used in the current study, and the experiment, are available in toluene. 

As shown in Figure 3 and Figure S1, the studied tautomers and transition states structures in both ground and excited state have been oriented in a local coordinate system in a way that the dipole moment vector coincides with the X-axis. This allows for facilitation of the effect of the EEF being applied in the direction of this axis and the neglect of the effect of the EEF being applied in direction of the other two axes. 

According to the general expectations, if the rotation of the structure around the Z-axis is not allowed, the EEF applied in the direction of X (i.e., in the direction of the dipole moment) stabilizes the structure and vice versa. As an example, the change in the energy of the corresponding ground-state tautomeric and transition-state structures as a function of the applied EEF and its direction is shown in Figure S2. As expected, the positive (stabilization) and negative (destabilization) effects of the EEF are related to the dipole moment of the corresponding structure—moderate in the case of **E**, for instance, and very dramatic in the case of the strongly polar **K** and **TS(KE-KK)**.

**Figure 4:**
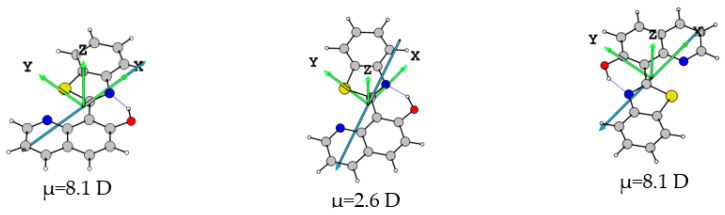
Orientation of **E** in respect to the local coordinate system when the rotation around the Z-axis is allowed and EEF is applied in the direction of the dipole moment (**left**) and against it (**right**). In the middle, the result with a molecule, fixed in respect to the Z-axis, is shown when the EEF is applied against the direction of the dipole moment. In this particular case the applied EEF is of 0.005 au.

The concept that the EEF, applied in the direction opposite to the structure dipole moment vector, destabilizes the structure is generally correct. However, the effects similar to those shown in Figure S2 can be considered in an infinite time scale only if the structure is fixed to the local coordinate system, which is the case of molecules deposed on a surface or being placed in an extremely viscous environment (polymer matrix). In solution, the structures placed in a stationary EEF, acting in the direction opposite to the dipole moment vector, should re-orient spontaneously in order to reduce this effect, and this happens in a relatively short time scale. This is actually demonstrated in Figure 4. In the left part of the figure, a structure is placed where the EEF is applied in the direction of the X-axis. The further stabilization of the structure is the net result. If the structure is constrained to the local coordinate system (in the current case this means that the rotation around Z-axis is not allowed) and the EEF is applied in the direction opposite to the X-axis, the structure is destabilized (Figure 4, in the middle). However, if the structure can compensate for the EEF effect by free rotation in the space, the orientation of the molecule leads to a dipole moment in the direction of the applied field as a net effect. This is shown in Figure 4, right. As a result of the free rotation in the space possible within the solution, the structures in Figure 4 left and right have exactly the same energy, although the initial direction of the EEF is exactly opposite. In other words, although the “opposite” EEF effect can be speculated, as is done for some tautomeric systems in solution [11,12,20], in actuality, the final stabilization of the structure in solution does not depend on the direction of the stationary EEF but on its strength and on the dipole moment of the corresponding structure. For this reason, we consider below only effects with a stationary EEF applied in the direction of the dipole moment vector. The same approach is applied in the study of the ESIPT of 2,5-bis(benzoxazol-2-yl)thiophene-3,4-diol in chloroform [21].

**Figure 5:**
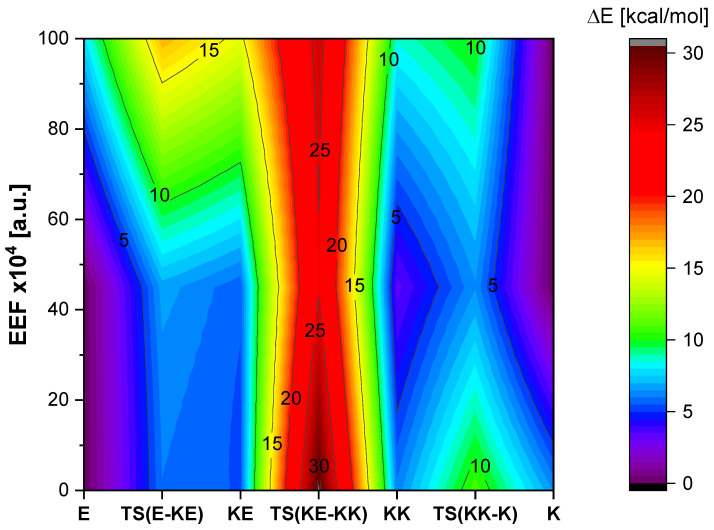
Contour map of the ground-state PESs of **HQBT** in toluene presented as a function of the EEF strength.

Bearing in mind the dipole moments of the ground (Figure 3) and excited-state (Figure S1) tautomers of **HQBT**, it is relatively easy to build some expectations about the EEF effect. In the ground state, the dipole moments of **TS(KE-KK)** and of the structures from the *PT region II* are substantially higher, which with the increasing strength of the EEF could lead to their stabilization and destabilization of the **E** and **KE** tautomers. The overall picture in the ground state is shown in Figure 5. In the absence of EEF, the E tautomer is the most stable and is only presented in solution. As seen from the Figure, with the increase in strength of the EEF, the **K**-tautomer becomes more stable, which finally leads to a full, ground state, switching from **E** to **K**. However, the process of relaxation of **K** back to **E**, when the EEF is turned off, is expected to be approximately the same rate because the barrier, determined by **TS(KE-KK)**, in respect to **K** remains approximately the same (from ~25 kcal/mol in the absence of EEF to ~26 kcal/mol at an EEF of strength 0.01 au). This result indicates that the EEF can be used as a stimulus for ground-state LRIPT-based switching if the relative polarities of the on and off state differ substantially. This is based on the thermodynamic distribution of the species and does not answer the question of how the LRIPT could happen as a mechanism.

**Figure 6:**
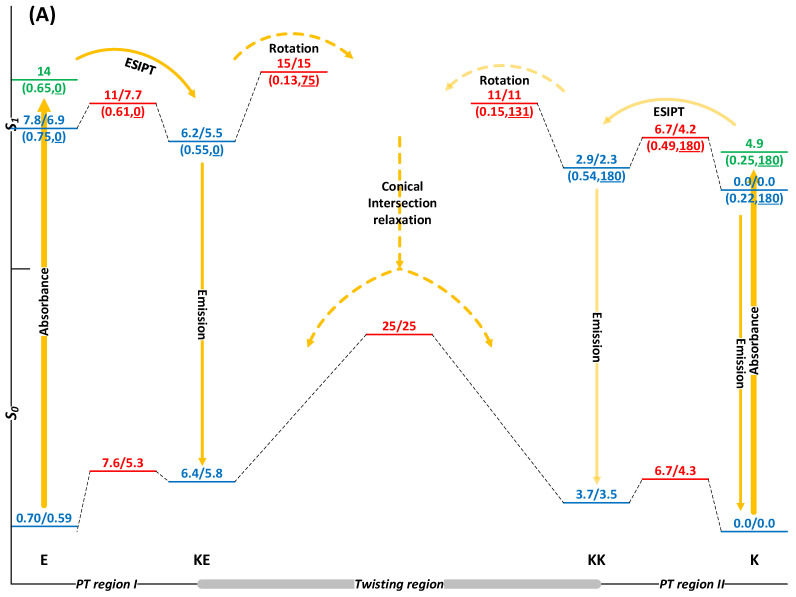

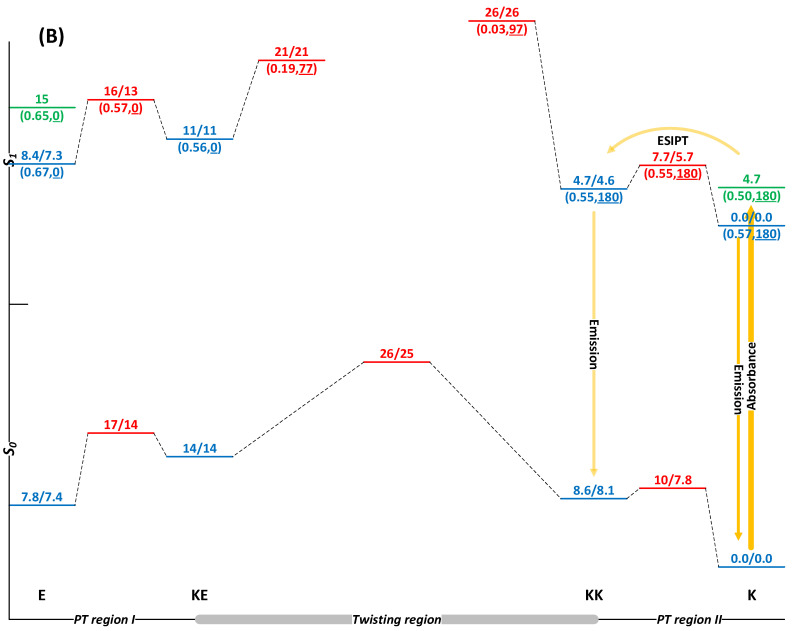
Potential energy surface (PES) of **HQBT** in toluene in ground and first singlet excited state at EEF of 0.005 (**A**) and 0.01 (**B**) au. The stationary forms are indicated in blue, the transition states—in red, the Frank–Condon state—in green. The numerical values present relative energy DE/relative Gibbs free energy DG and are given in kcal/mol units. In the excited state, the oscillator strength and the angle of rotation of the rotor against the stator are given in brackets, the latter is underlined.

To go into detail, at an EEF in the range of 0.004–0.005 au, the **E** and **K** tautomers become equally stable, as seen in Figure 5 and Figure 6, top. This means that in a relatively short period, the energy of **TS(KE-KK)** is reduced substantially, and after the EEF has been applied, spectral evidence for shifting the equilibrium towards **K** should be available with the appearance of a red-shifted shoulder around 440–410 nm (see Figure S3). The existence of both **E** and **K** simultaneously could lead to the appearance of the emission coming from K* along with existing ESIPT-based emission from KE*. As is seen, the changes in the excited state are not substantial, but the relative stabilization of KK* and the reduction in the TS(KK*-K*) might lead to an additional weak ESIPT emission. In general, the co-existence of **E** and **K** in the ground state makes the system unsuitable for clean switching, Clean switching is considered as a switching process starting from the pure off state (in this particular case—**E**) and ending to the pure on state (here—**K**) when a suitable external stimulus is applied. Upon irradiation, depending on the possibility for ESIPT from K* to KK*, two scenarios are possible. If there is such ESIPT followed by rotation of the rotor in respect to the stator, the irradiation could cause slight fluctuations in the proportion between **E** and **K**, because two opposite switching processes could be in action (from **E** to **K** and from **K** to **E**). However, the increase in the EEF strength stimulates the intramolecular charge separation and the breakage of the intramolecular hydrogen bonding in the excited state, and the twisting after that could become more energy demanding and less efficient. In such a case, the parallel mutually independent PT processes in *PT regions I* and *II* could dominate. In the opposite case, if there is no ESIPT, accumulation of **K** could be expected ending in an ideal case of the full disappearance of **E**. 

The further increase in the EEF leads to further stabilization of the structures in the *PT region II* as shown in Figure 6, bottom. At an EEF of 0.01 au, the **K** tautomer should only be presented in solution some period after turning on the field, thus performing a clean switching from **E**. In this way, we show that a complete shift of the tautomeric state of **HQBT** is possible without applying irradiation or changing the solvent. However, comparing the absorption spectra of **E** and **K** before (Figure 2) and after (Figure S4) the EEF-based switching, it seems that the switching event would be hard to detect. The increase in the strength of the EEF leads to a decrease in the HOMO-LUMO gap in **E** (see Table S1), causing a slight red shift. Exactly the opposite effect is observed in the case of **K**, where the oscillator strengths are rising in addition. All this together leads to a situation where the spectra of **E** and **K** overlap in a way that it is difficult to distinguish. This is evident in Figures S4 and S5. No substantial changes in the excited state could be expected at this EEF upon irradiation, for the reasons discussed above, which makes the photoinduced population of **E** from **K** highly unlikely. As is seen, the K* tautomer becomes more and more stabilized in respect to KK*, which reduces the possibility for ESPIT to KK* and consecutive rotation to the conical intersection region. In this case the only way to achieve the restoration of the originally existing **E** form is to turn off the EEF.

### 2.3. Theoretical Methodology

Quantum-chemical calculations were performed using the Gaussian 16 C.01 program suite [22]. All structures (in both ground and excited state, in absence or presence of EEF) were optimized without restrictions, using tight optimization criteria and an ultrafine grid in the computation of two-electron integrals and their derivatives. The true minima were verified by performing frequency calculations in the corresponding environment. The implicit solvation was described using the Polarizable Continuum Model [23] (the integral equation formalism variant, IEFPCM, as implemented in Gaussian 16). The transition states were estimated using the STQN method [24] and again verified by performing frequency calculations in the corresponding environment. The effect of the external electric field was modelled by adding a finite electric dipole field as implemented in Gaussian 16. The internal coordinate system was oriented in a way that the X-axis coincides with the dipole moment vector.

The M06-2X [25,26] functional with the def2TZVPP [27] basis set was used for the structure optimizations in the ground state. The use of M06-2X provides very good predictability in the ground-state [17,28,29,30,31,32] tautomeric composition in tautomeric compounds and proton cranes in solution as well as the E/Z isomerization ratio in some rotary switches.

The TD-DFT method [33,34,35] was used for the singlet excited-state optimizations. For consistency with the ground-state calculations, the M06-2X functional with the desf2TZVPP basis set was again used for the optimizations. Previously, we have shown [17] that in the case of **HQBT**, the results for the excited-state PESs, obtained by M06-2X and CAM-B3LYP, are essentially the same. In addition, the DFT results for both ground and excited state were validated [17] by using domain-based local pair natural orbital coupled cluster singles and doubles and perturbative triple excitations (DLPNO-CCSD(T) [36]), and domain-based local pair natural orbital-similarity transformed equation of motion coupled cluster singles and doubles (DLPNO-STEOM-CCSD [37]), respectively.

Bearing in mind that M06-2X systematically underestimates the absorption band positions [38], the UV–Vis spectral data were predicted by using the B3LYP [39] functional (def2TZVPP basis set) using the M06-2X optimized ground-state geometries. The spectra were simulated according to the methodology described previously by us [40] by using single Gauss shape band half-band width (${\Delta \nu }_{1/2}$) of 3000 cm^−1^.

## 3. Conclusions

We have shown theoretically that in the case of **HQBT**, the increase in the strength of the EEF can lead to complete ground-state switching from the enol to the end keto tautomer **K**. This is a result of the much larger dipole moment of **K**. In other words, the increase in the EFF mimics the change in the solvent with another, with continuously increasing polarity. This could be of practical importance in any equilibrium process. Since the use of EEF leads to strong stabilization of K* and makes it more difficult to break the intramolecular hydrogen bonding, photochemical switching from **K** to **E** could not be expected.

## Data Availability

The data presented in this study are available in the article and Supplementary Materials.

## Supplementary Materials

The following are available online at https://www.mdpi.com/article/10.3390/molecules29153506/s1, Figure S1: Direction of the dipole moment vectors (in light blue) of the first singlet excited state tautomeric forms and transition states of HQBT in respect of the local coordinate system (in green). The atoms are given as follows: O (red), N (blue), S (yellow), C (grey), H (white). The values of the dipole moments and twisting angle are given for information. Figure S2: Relative stabilization (relative energy, up, relative Gibbs free energy, down) of the tautomers of HQBT and the transition states between them in toluene as a function of the strength and direction of the EEF in respect of the X-axis. Figure S3: Theoretically predicted spectra of the tautomers of HQBT in toluene in presence of EEF of 0.005 au, given as simulated absorption curves (left) and transitions with corresponding oscillator strength (right). Figure S4: Theoretically predicted spectra of the tautomers of HQBT in toluene in presence of EEF of 0.01 au, given as simulated absorption curves (left) and transitions with corresponding oscillator strength (right). Figure S5. Evolution of the spectra to be measured as a function of the EEF. Each spectrum is a function of the fractions of the individual tautomers and their individual spectra. Table S1. Theoretically predicted long-wavelength absorption bands, with corresponding oscillator strengths, of the tautomeric forms of HQBT in toluene at different strength of the EEF.
