# Supplementary material for: Stationary External Electric Field—Mimicking the Solvent Effect on the Ground-State Tautomerism and Excited-State Proton Transfer in 8-(Benzo[d]thiazol-2-yl)quinolin-7-ol
Source: Molecules. 2024 Jul 26;29(15):3506. doi: 10.3390/molecules29153506 (PMC11313717; doi:10.3390/molecules29153506)
Supplement: Supplementary file 1 [file molecules-29-03506-s001.zip › molecules-3124621-supplementary.pdf]

Supporting information

Stationary External Electric Field—Mimicking the Solvent Effect on the Ground-State Tautomerism and Excited-State Proton Transfer in 8-(Benzo[d]thiazol-2-yl)quinolin-7-ol

Lidia Zaharieva, Ivan Angelov and Liudmil Antonov *

Institute of Electronics, Bulgarian Academy of Sciences, Sofia 1784, Bulgaria; zaharievalidia@gmail.com (L.Z.); ipangelov@gmail.com (I.A.)
*Correspondence: lantonov@tautomer.eu

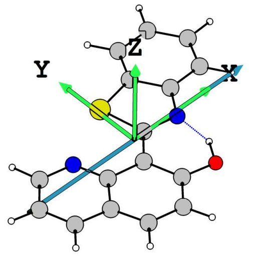

E*, μ= 3.5 D, α=0 o

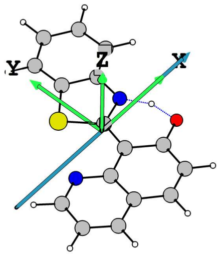

TS(E*-KE*), μ= 2.5 D, α=0 o

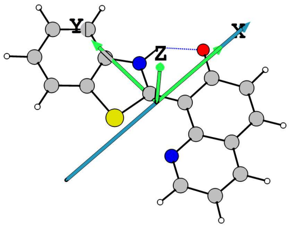

KE*, μ= 2.9 D, α=0 o

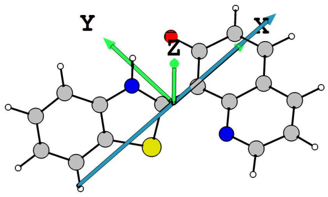

TS(KE*-KE'*), μ= 5.2 D,

α=49 o

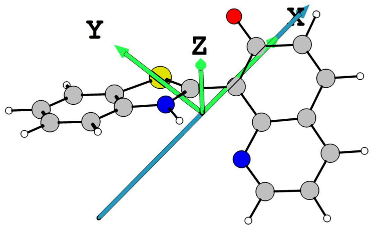

TS(KK*-KK'*), μ= 7.2 D,

α=132 o

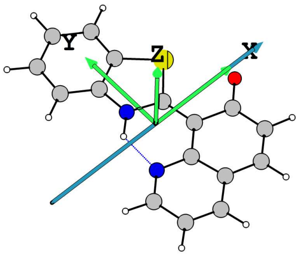

KK*, μ= 5.8 D, α=180 o

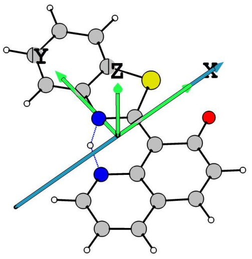

TS(KK*-K*), μ= 7.1 D,

α=180 o

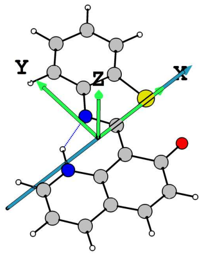

K*, μ= 9.7 D, α=180 o

Figure S1. Direction of the dipole moment vectors (in light blue) of the first singlet excited state tautomeric forms and transition states of **HQBT** in respect of the local coordinate system (in green). The atoms are given as follows: O (red), N (blue), S (yellow), C (grey), H (white). The values of the dipole moments and twisting angle are given for information.

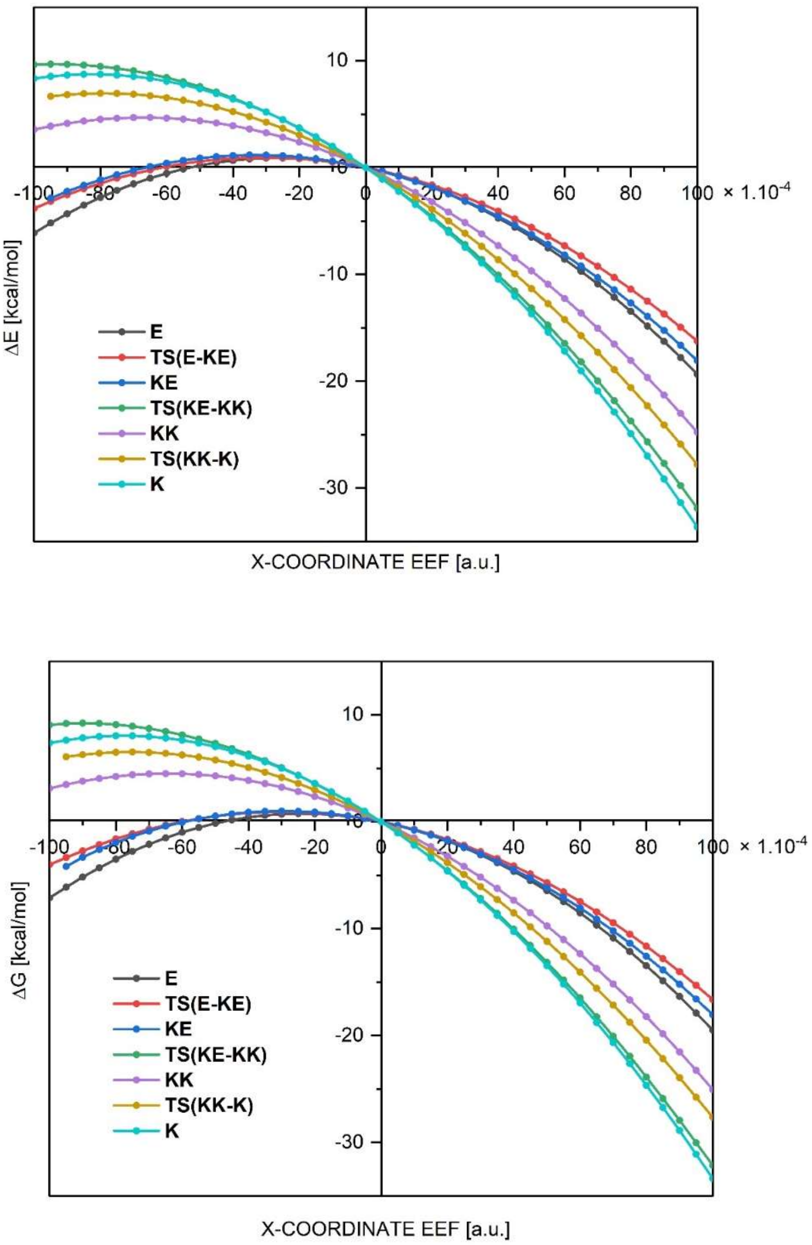

Figure S2. Relative stabilization (relative energy, up, relative Gibbs free energy, down) of the tautomers of **HQBT** and the transition states between them in toluene as a function of the strength and direction of the EEF in respect of the X-axis.

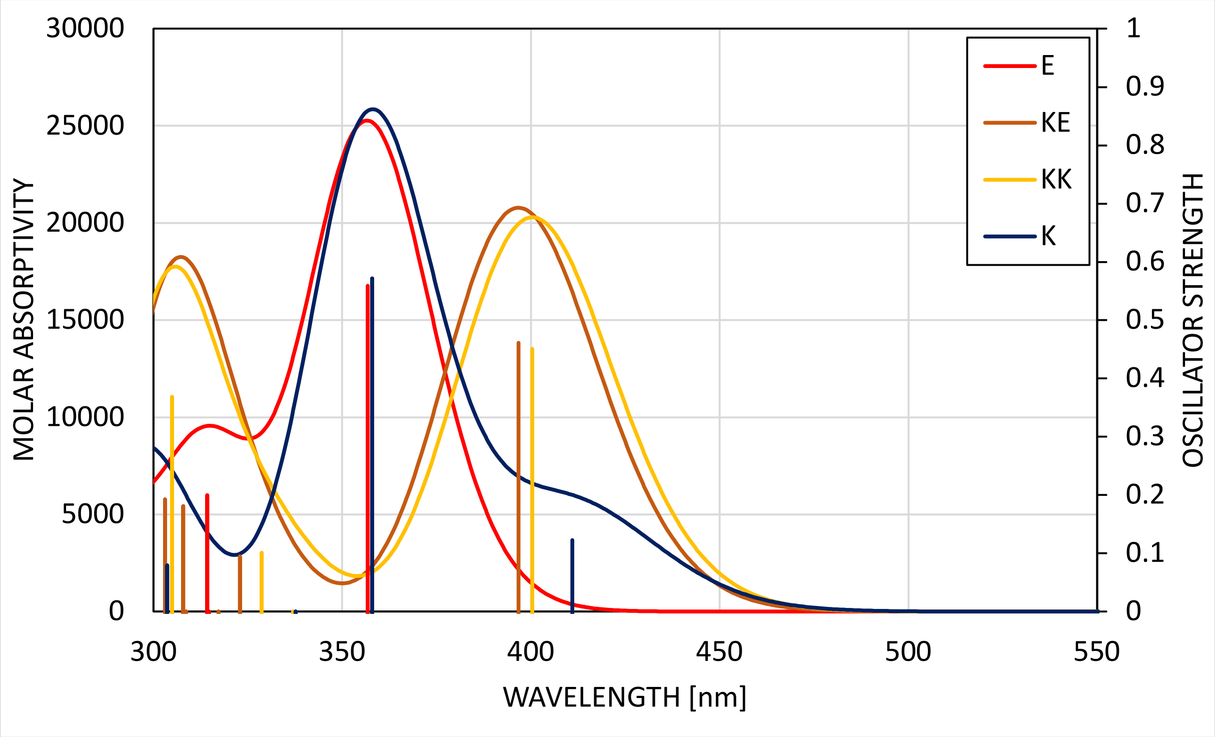

Figure S3. Theoretically predicted spectra of the tautomers of **HQBT** in toluene in presence of EEF of 0.005 au, given as simulated absorption curves (left) and transitions with corresponding oscillator strength (right).

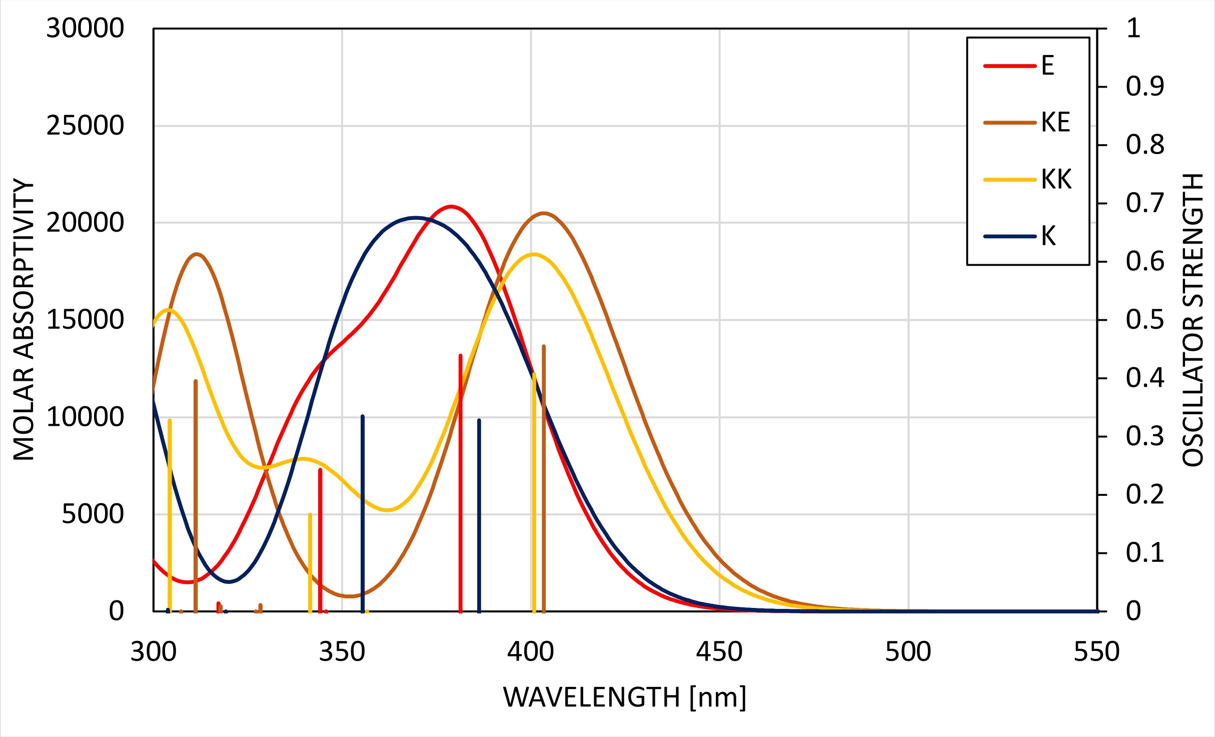

Figure S4. Theoretically predicted spectra of the tautomers of **HQBT** in toluene in presence of EEF of 0.01 au, given as simulated absorption curves (left) and transitions with corresponding oscillator strength (right).

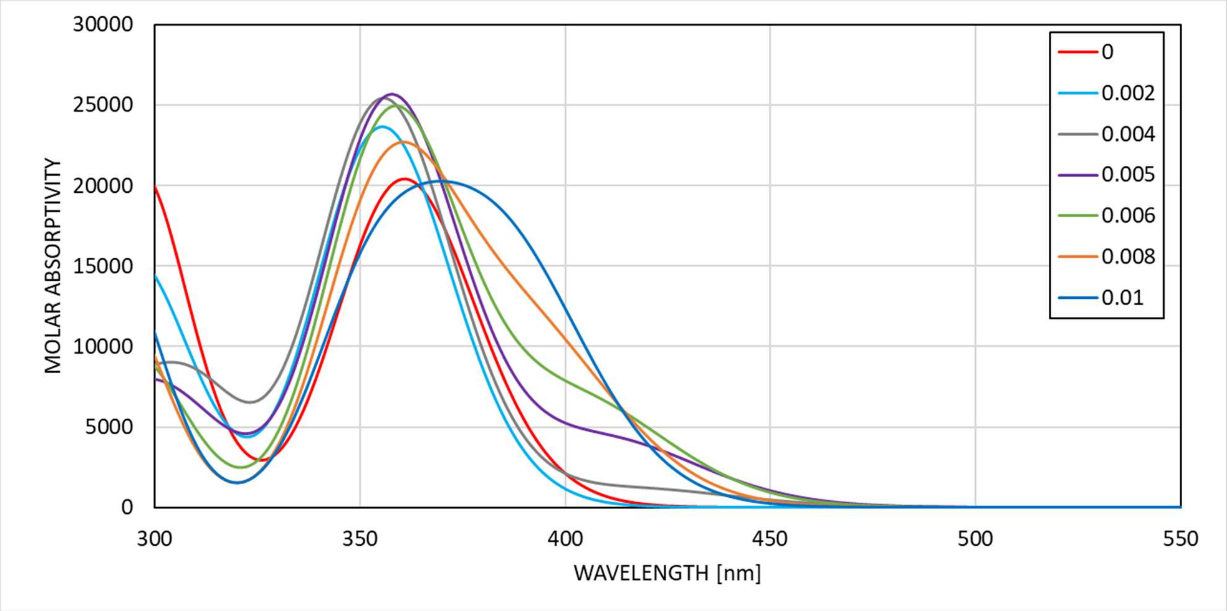

Figure S5. Evolution of the spectra to be measured as a function of the EEF. Each spectrum is a function of the fractions of the individual tautomers (see the Table below) and their individual spectra.

EEF	**E** [%]	**KE** [%]	**KK** [%]	**K** [%]
0	100	0	0	0
0.002	100	0	0	0
0.004	77	0	0	23
0.005	27	0	0	73
0.006	3	0	0	97
0.008	0	0	0	100
0.01	0	0	0	100

Table S1. Theoretically predicted long-wavelength absorption bands, with corresponding oscillator strengths, of the tautomeric forms of **HQBT** in toluene at different strength of the EEF.

**X-coordinate EEF** ×1.10^{-4} [a.u.]	**E**		**KE**		**KK**		**K**	
	λ_{max} [nm]	*f*	λ_{max} [nm]	*f*	λ_{max} [nm]	*f*	λ_{max} [nm]	*f*
100	381	0.44	403	0.45	401	0.41	386	0.33
90	374	0.47	402	0.46	403	0.46	389	0.26
80	368	0.51	400	0.46	403	0.46	394	0.21
70	363	0.53	399	0.46	402	0.46	399	0.17
60	359	0.55	398	0.46	401	0.45	405	0.14
50	357	0.56	397	0.46	400	0.45	411	0.12
40	355	0.56	396	0.46	400	0.45	418	0.11
30	355	0.55	395	0.47	399	0.44	425	0.10
20	355	0.53	393	0.47	398	0.44	432	0.09
10	357	0.49	393	0.47	398	0.44	438	0.08
0	361	0.45	392	0.48	398	0.43	448	0.08
